# Development of a Novel Surgical Method for Endoscopic Flexor Tendon Repair: Stage 0 (Preclinical) Cadaveric Case Report and Study Protocol Following the IDEAL-D Framework

**DOI:** 10.7759/cureus.85109

**Published:** 2025-05-30

**Authors:** Takeru Yokota, Takuya Kameda, Nobuyuki Sasaki, Miho Sekiguchi, Yoshihiro Matsumoto

**Affiliations:** 1 Department of Orthopaedic Surgery, Fukushima Medical University School of Medicine, Fukushima, JPN; 2 Department of Orthopaedics, Fukushima Red Cross Hospital, Fukushima, JPN

**Keywords:** flexor tendon injury, hand surgeon, minimal invasive surgeon, surgical endoscopy, tendon surgery

## Abstract

Flexor tendon injuries of the hand are challenging to treat, with open surgical repair causing tissue damage, postoperative edema, and range-of-motion limitations. Minimally invasive endoscopic techniques may address these issues, but no reports describe endoscopic tendon suturing. This IDEAL-D Stage 0 (Preclinical) study presents a cadaveric case report and research protocol for a novel endoscopic flexor tendon repair technique. The case report used the left ring finger of an 88-year-old male cadaver, employing a loop suture technique under endoscopic guidance to visualize and suture tendon ends. Sutures facilitated tendon gliding, withstood 5 N tension, and caused no neurovascular injuries. The protocol involves five cadaver specimens from Fukushima Medical University, excluding those with deformities or prior hand injuries. The primary outcome is the success rate of endoscopic repair, defined as proper tendon alignment, tension, and integrity without complications. Secondary outcomes include suturing time, gap distance, complications, a proposed tendon injury classification system, and a surgical technique video. Results will be analyzed using descriptive statistics with 95% confidence intervals. This technique may offer a minimally invasive, precise solution for flexor tendon injuries.

## Introduction

Flexor tendon injuries are common, occurring in 4.83 per 100,000 individuals [1]. These injuries are often caused by trauma, such as lacerations, and can significantly affect patients’ ability to perform daily activities [2]. Flexor tendon injuries are associated with a substantial impact on quality of life, often resulting in reduced hand function, pain, and disability [3,4].

Appropriate treatment is essential for flexor tendon injuries; however, their management is often challenging [4]. The standard treatment approach has traditionally been open surgical repair, which involves direct exposure of the tendon for suturing [4,5]. However, this open approach can cause significant soft tissue damage, increasing postoperative edema and restricting joint range of motion (ROM) [5]. This highlights the importance of developing minimally invasive surgical (MIS) techniques, with endoscopy emerging as an effective tool [6].

Multiple studies have reported open approaches for flexor tendon repair; however, studies on endoscopic techniques remain limited [7-9]. Certain studies reported hand surgeons attempting to apply endoscopic approaches in flexor tendon repair, such as locating and retrieving tendon ends using endoscopy [7-9]. However, to the best of our knowledge, no reports of tendon suturing performed entirely under endoscopy have been published.

The IDEAL-D framework [10-12], tailored for device innovations, supports preclinical Stage 0 studies like this cadaveric evaluation [13,14]. This framework provides a structured approach that acknowledges the unique challenges inherent in surgical innovations, such as reliance on the skill and judgment of the individual surgeon and customization of interventions for each patient. This report presents a Stage 0 (preclinical) cadaveric case report and study protocol for a novel endoscopic tendon repair technique following the IDEAL-D framework.

## Case presentation

This cadaveric case report, conducted under the IDEAL-D framework’s Stage 0 (Preclinical), evaluates the feasibility of a novel endoscopic tendon repair technique in Zone 1 flexor tendon injuries.

The cadaver was an 88-year-old male. No significant trauma or deformities were observed on the hand or fingers of the cadaver.

The index and middle fingers were used to create a tendon injury model and examine the appearance of the tendon stump under endoscopy. Meanwhile, the left ring finger was used to verify the technique of endoscopic tendon repair.

**Figure 1:**
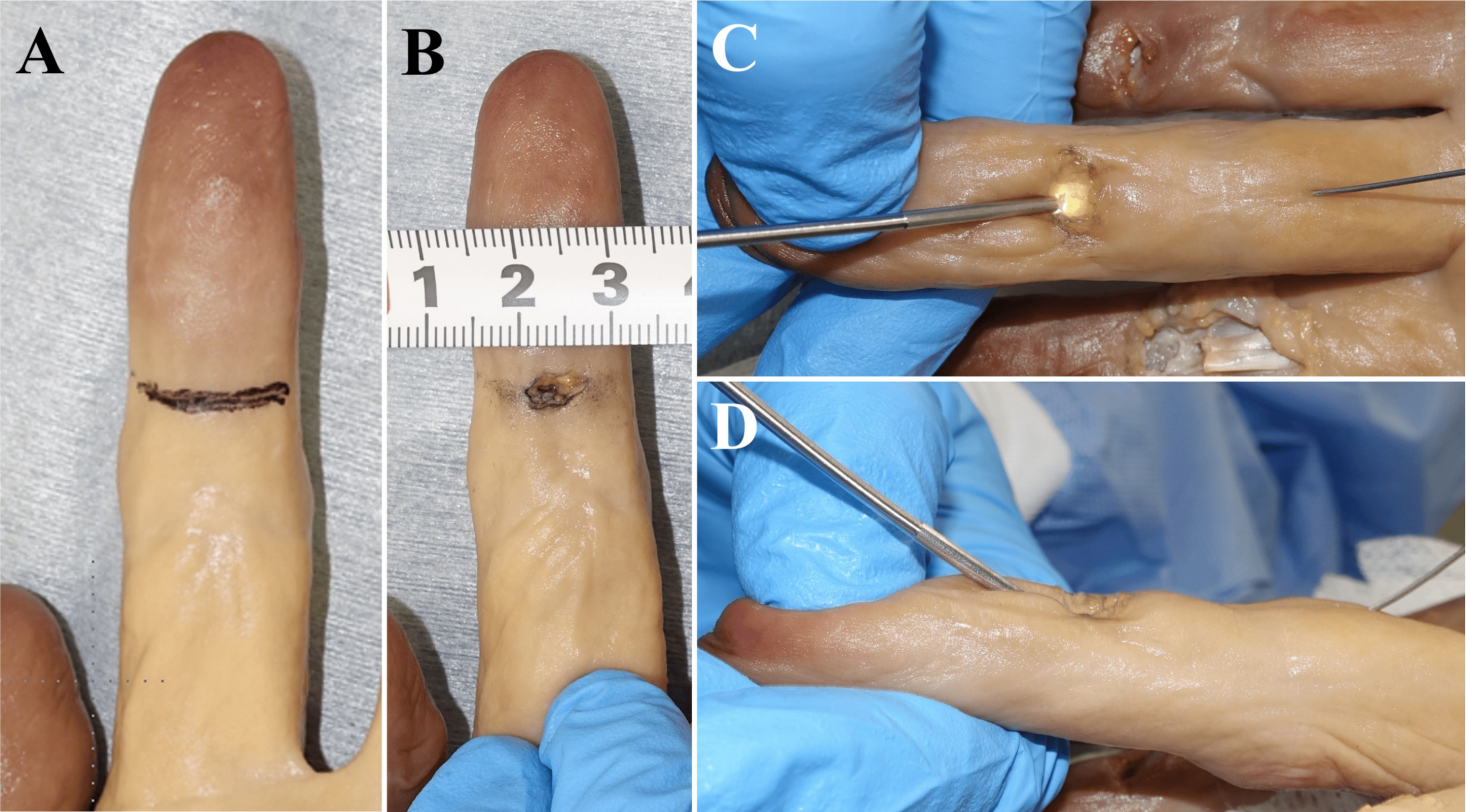
Creation of Flexor Tendon Injury Model and Endoscopic Confirmation of the Flexor Digitorum Profundus Tendon A: An incision line was designed at the proximal one-third of the middle phalanx. B: A 5 mm wide transverse incision was made. C and D: The endoscope was inserted from the distal to the proximal side (C shows the palmar view, D shows the lateral view).

A flexor tendon injury model using the left index and middle fingers was created by making a 5 mm transverse skin incision on the distal one-third of the left finger's middle phalanx (Figure 1A, 1B). The tendon sheath and the flexor digitorum profundus (FDP) were sharply incised using a #11 scalpel at the incision site, creating a zone I injury [15]. An endoscope, NanoScope® (Arthrex, Naples, FL), was subsequently inserted through the incision into the tendon sheath (Figure 1C, 1D). The central side of the tendon sheath was visualized using the endoscope, allowing confirmation of the end of the FDP.

Procedure of endoscopic tendon suture

**Figure 2:**
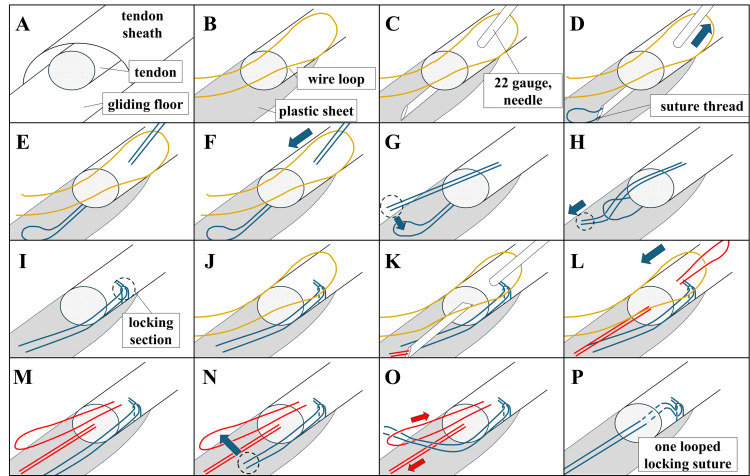
Illustration of the Procedure for Endoscopic Flexor Tendon Suturing A: Overview of the tendon, tendon sheath, and glide floor. B-E: Passing the suture thread through the tendon. F-I: Creating the lock section of the suture thread around the tendon. J-L: Guiding the guiding thread through the tendon. M-O: Using the guiding thread to pass the suture thread through the tendon. P: Completion of the one-looped locking suture. The yellow line represents a metal wire used for guiding the thread, blue line represents the suture thread, and red line represents the guiding thread.

**Figure 3:**
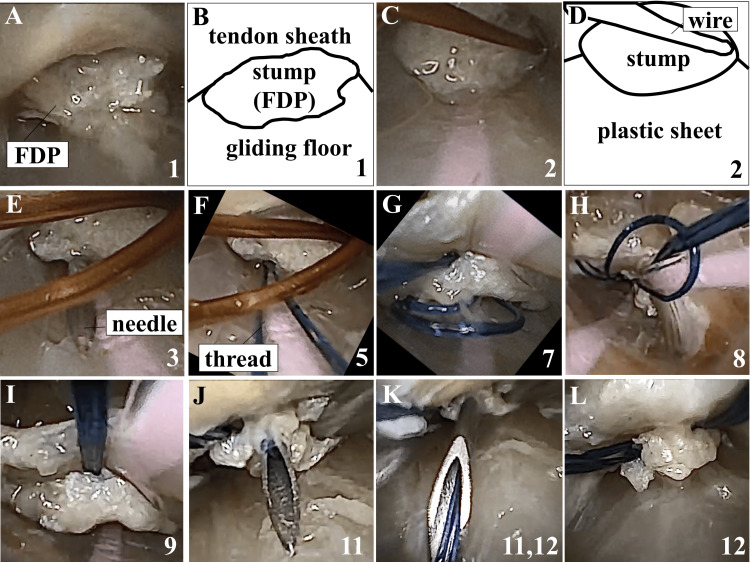
Endoscopic Views and Illustrations of the Endoscopic Flexor Tendon Suturing Procedure The numbers indicated in this figure correspond to the steps described in the "Procedure of Endoscopic Tendon Suture" section of the Case Presentation in the main text. B is the illustration corresponding to A, and D is the illustration corresponding to C. FDP: Flexor digitorum profundus.

Subsequently, the left ring finger was used to attempt endoscopic flexor tendon repair. Suture of the tendon was attempted under endoscopic guidance, opting for a one-looped suture method (Figure 2). The suturing technique is explained below, with steps 1-16 corresponding to the numbered illustrations in Figure 3.

1. This illustrates the overview before inserting the endoscope. The tendon stump is located within a tube surrounded by the tendon sheath and gliding floor (Figure 2A).

2. A plastic sheet was inserted beneath the tendon within the sheath to avoid damaging the gliding bed with the suture needle. Simultaneously, a wire loop was placed above the tendon to guide the suture thread for subsequent tendon suturing (Figure 2B).

3. A 22-gauge needle containing 4-0 nylon thread was inserted from the skin to the deep layer, passing through the wire loop placed above the tendon. The needle was then advanced from the external surface of the tendon into its interior and passed through to emerge on the opposite external surface of the tendon. This step is crucial for performing lock suturing (Figure 2C).

4, 5. The suture thread stored inside the needle was pushed out through the needle tip, and the needle was removed. This allows the looped thread to pass through the tendon in Step 5 (Figure 2D, 2E).

6. The wire loop was pulled out to retrieve the opposite end of the looped thread (Figure 2F).

7-9. The opposite end of the thread passed through the loop of the thread, creating a locking section around the tendon (Figure 2G-2I).

10-13. The procedures in Steps 2-6 were repeated to guide the suture thread into the tendon. The guiding thread was positioned using the needle and wire, allowing the suture thread to pass through the tendon (Figure 2J-2M).

14-16. The end of the suture thread passed through the loop of the guiding thread. The suture thread was pulled through the tendon by pulling the opposite end of the guiding thread, completing the one-looped locking suture (Figure 2N-2P).

Photographs of the procedure under endoscopic guidance from Step 1 to Step 12 are shown in Figure 3. Video 1 provides additional information, showing the locking section creation in Steps 7-9 and the thread withdrawal in Steps 6, 12-13, and 15-16.

**Video 1:** Demonstration of suturing technique, gliding test, and 5 N tensile load test This video provides an overview of specific steps in the suturing procedure, including the creation of the locking section (Steps 7-9) and thread withdrawal (Steps 6, 12-13, and 15-16). Moreover, the video demonstrates tendon gliding within the tendon sheath after suturing (gliding test) and the tensile test results. For the tensile test, 500 mL saline solution was vertically suspended to simulate a 5 N tensile load on the suture threads.

**Figure 4:**
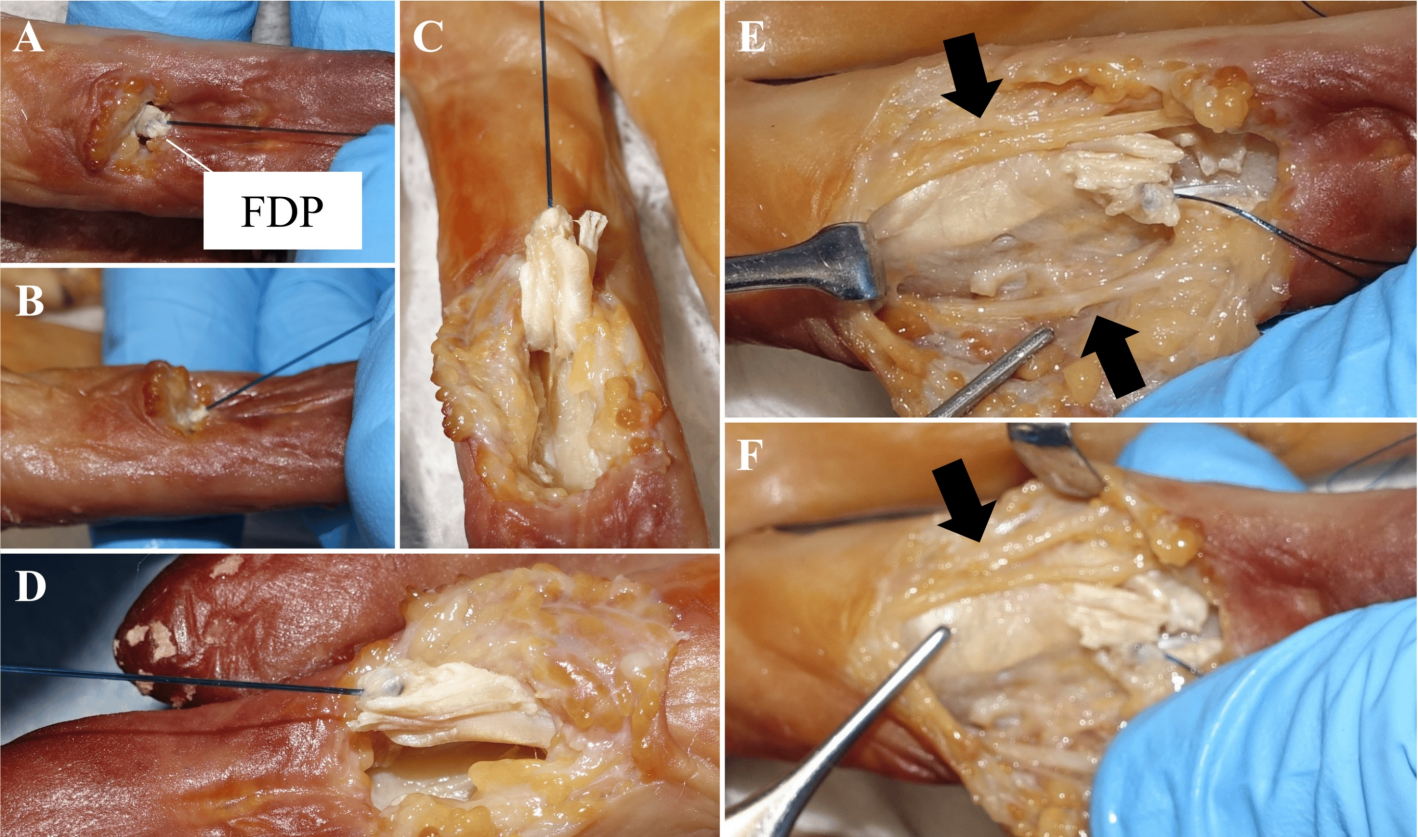
Photographs After Completing the One-Looped Locking Suture A, B: The suture site of the FDP was pulled, and the tendon was retracted to the level of the injury (A: palm side, B: side view). C, D: The soft tissues, including the tendon sheath, were incised to expose the tendon substance. E, F: The neurovascular bundle was evaluated for damage, and no damage was observed. The black arrow indicates the neurovascular bundle. FDP: flexor digitorum profundus.

After completing this suture, manual traction was applied to confirm the gliding of the tendon (Figure 4A, 4B). Subsequently, the soft tissue and tendon sheath were incised to expose the sutured area, confirming that the suturing procedure had been successfully performed (Figure 4C, 4D). The soft tissue and tendon sheath were subsequently dissected to assess the damage to the neurovascular bundles (Figure 4E, 4F). Finally, continuous traction on the suture end with 500 mL of saline was applied to simulate a 5 N tension test to confirm the completion of tendon repair.

Video 1 additionally demonstrates the tendon glide within the tendon sheath after suturing and the tensile test results. In this test, 500 mL of saline solution was continuously suspended vertically from the two strands of suture thread extending from the tendon stump to simulate a 5 N tensile load on the threads. The choice of 5 N as the tensile load was based on the assumption that flexor tendons experience approximately 10 N of tension during active motion [16]. The tensile load on each suture thread distributes to approximately 3.3-5 N when employing 2-3 one-loop sutures. Therefore, a 5 N tensile load was selected for this simulation. No failure of the sutured area was observed after the tensile test.

Study protocol

Objectives

The primary objective of this study is to develop an endoscopic tendon repair method and assess its feasibility using cadaveric models. Secondary objectives include assessing the safety and efficacy of the technique, evaluating surgical difficulty, and examining suitable tendon injury classifications for this endoscopic technique.

Study Significance

The usefulness of MIS techniques in flexor tendon repair has been reported; however, established methods have not yet been developed. The development of endoscopic-assisted MIS techniques is expected to expand treatment options based on individual circumstances. Particularly, endoscopic tendon repair has the potential to minimize tissue damage, reduce postoperative edema, and improve joint ROM, thereby improving functional recovery.

Study Methods and Analysis

This study corresponds to Stage 0 (preclinical) of the IDEAL-D framework [10-12]. This development study was reviewed and approved by the Ethics Committee of Fukushima Medical University in Japan (CRB2200002) (Approval No. REC2023-115). The study protocol was registered in the Japan Registry of Clinical Trials (J-RCT) (No. jRCT1022230036), and the study will be conducted in accordance with the principles of the Declaration of Helsinki.

Study Design

**Figure 5:**
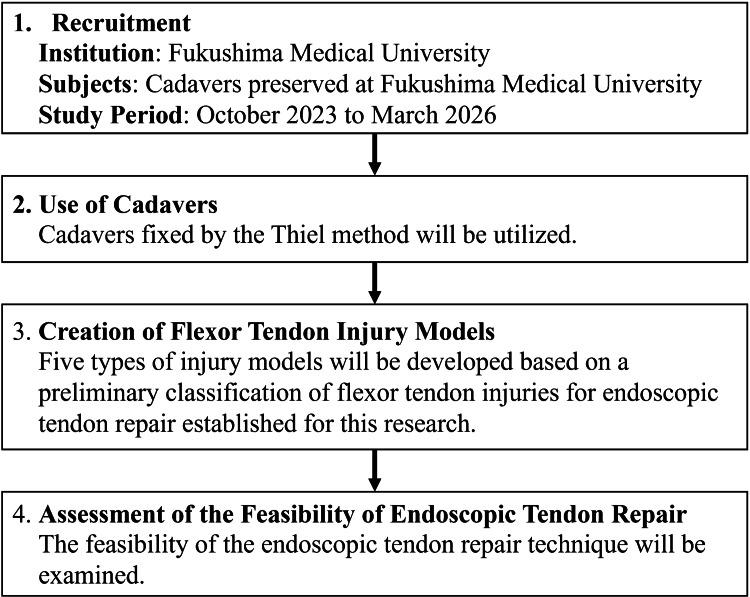
Illustration of the Schema of the Development Study

This study involves the development of a novel surgical technique using cadaveric models. Flexor tendon injury models in cadavers will be used to develop the endoscopic tendon suturing method. The schema of the study is shown in Figure 5.

Participants

Cadaveric specimens preserved at the Fukushima Medical University will be used for this study, with all donors voluntarily donating their bodies.

The exclusion criteria include cadavers with hand deformities or significant trauma, including old injuries. These criteria were established based on the anticipated difficulty in obtaining standard visual fields for tendon suturing under endoscopy in cadavers with significant hand deformities or trauma.

**Figure 6:**
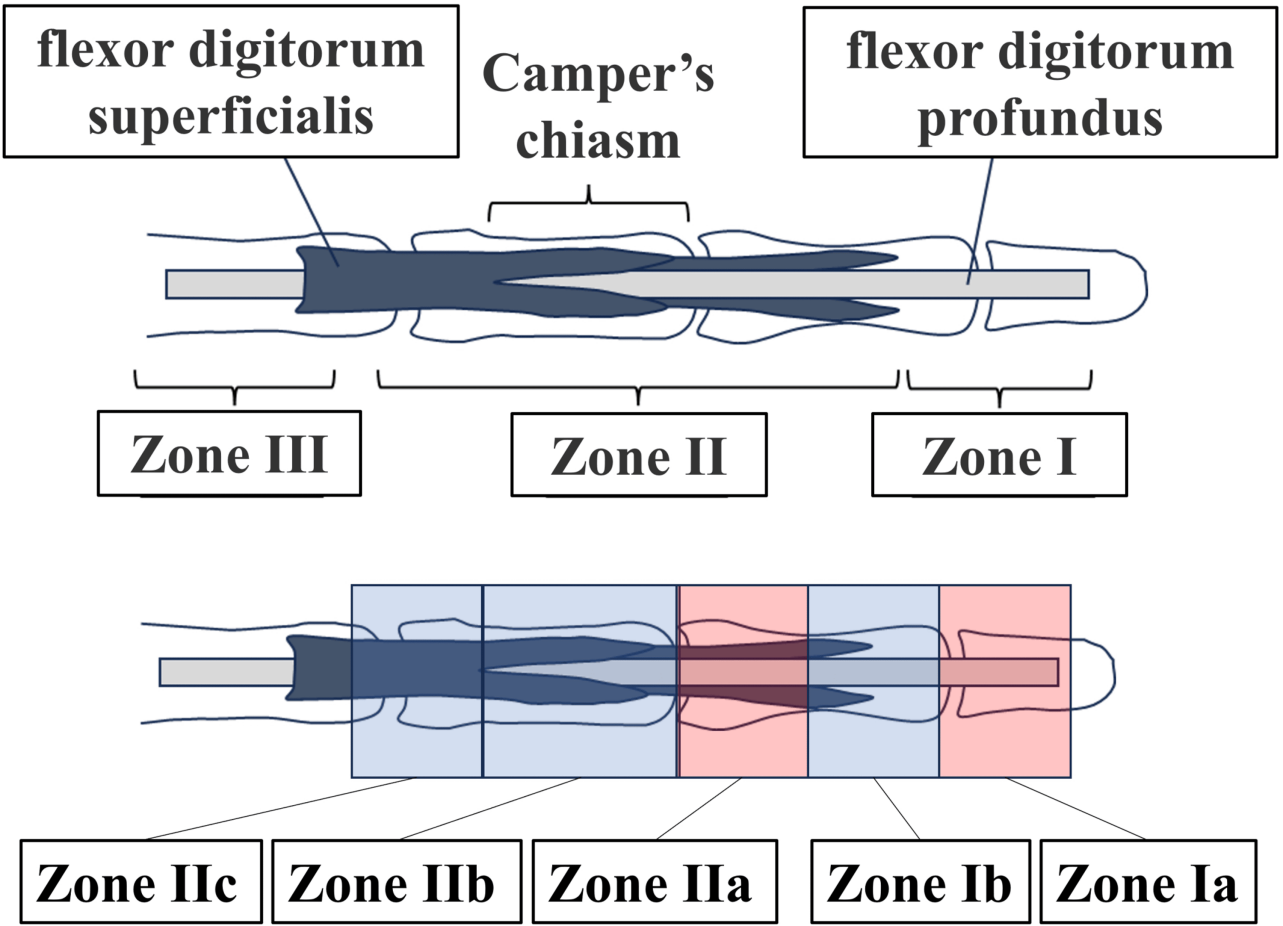
Proposed Classification for Endoscopic Flexor Tendon Injury (Yokota Classification) The upper section presents the traditional zone classification for flexor tendon injuries, divided into Zones I to V. Zone I is defined as the portion distal to the midpoint of the middle phalanx, Zone II extends from the midpoint of the middle phalanx to the MP joint, and Zone III extends to the midpoint of the MP. We created a classification for endoscopic flexor tendon repair for our study, subdividing Zone I into Ia and Ib and Zone II into IIa, IIb, and IIc. Herein, "a" indicates that <10 mm of tendon remnant remains at the cut end of either the FDP or FDS, whereas "c" signifies that the injury is located proximal to the camper’s chiasm. MP: metacarpal; FDP: flexor digitorum profundus; FDS: flexor digitorum superficialis.

**Figure 7:**
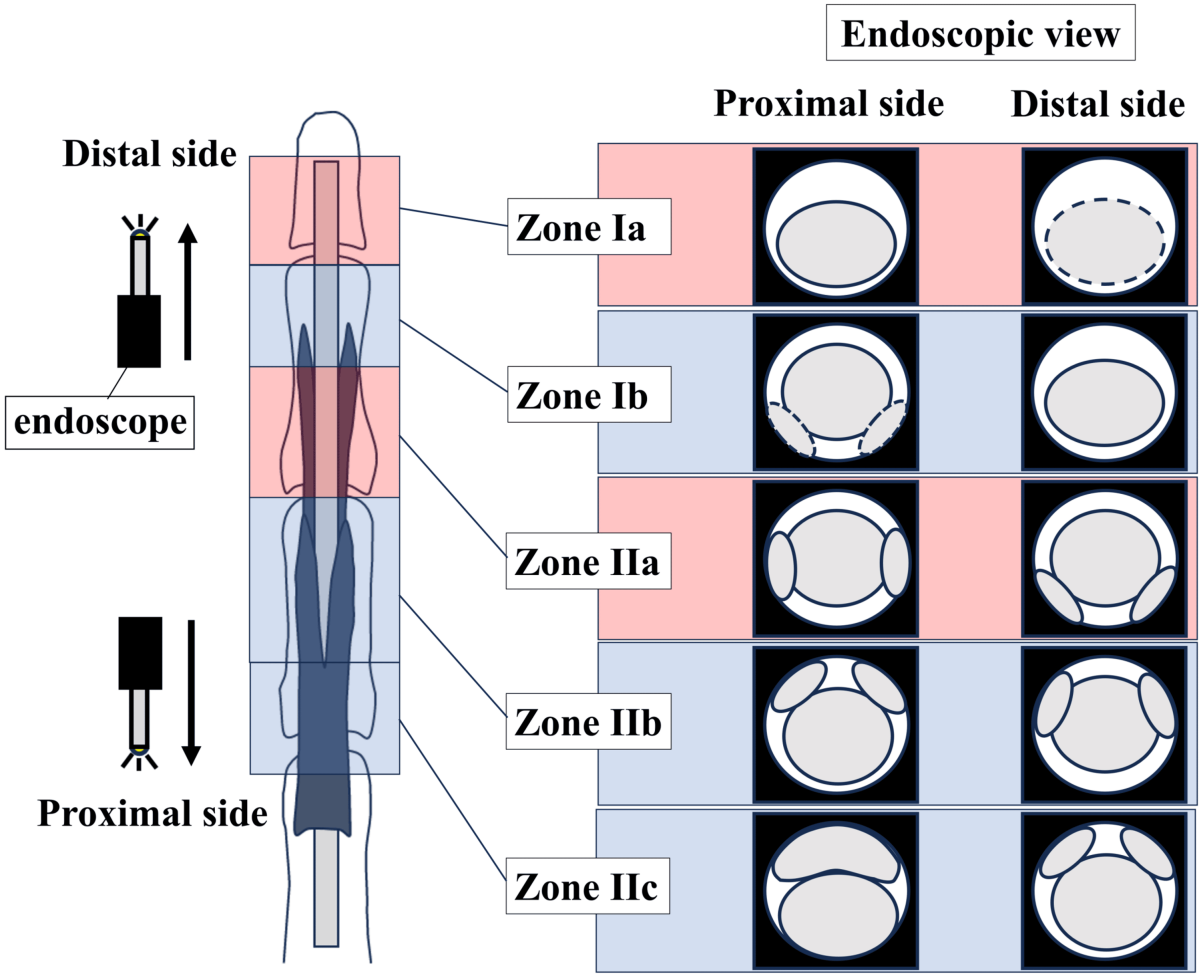
Endoscopic Views Corresponding to the Proposed Classification for Endoscopic Flexor Tendon Injury This figure illustrates the endoscopic view corresponding to the tendon injury classification for endoscopic flexor tendon repair. The white circle in the "endoscopic view" indicates the endoscope field, whereas the gray area represents the cross-section of the tendon injury site. The dashed lines indicate the possibility that the tendon substance may not be visible due to an avulsion rupture.

The minimum sample size for this study is five cadavers, corresponding to 40 fingers [5 cadavers × 8 fingers (excluding the thumbs, totaling four fingers per hand)]. This sample size was established based on a preliminary classification of flexor tendon injuries for endoscopic tendon repair that was developed for this research (Figures 6, 7, Table 1), which modifies the traditional zone classification of flexor tendon injuries [15].

**Table 1:** Revised Zone Classification for Endoscopic Tendon Repair With Corresponding Injury Types and Repair Techniques *A revised classification for endoscopic tendon suturing was developed based on the traditional zone classification of flexor tendon injuries. This table describes the revised zone classification, along with corresponding injury types and repair techniques. FDS: flexor digitorum superficialis; FDP: flexor digitorum profundus; N.A.: not applicable.

Classification of Tendon Injuries in Endoscopic Tendon Repair*	Injury Type (FDS)	Injury Type (FDP)	Repair Technique (FDS)	Repair Technique (FDP)
Zone Ia	N.A.	Avulsion or near-avulsion rupture	N.A.	Pull-out or suture anchor
Zone Ib	N.A.	Rupture	N.A.	End-to-end suture
Zone IIa	Avulsion or near-avulsion rupture	Rupture	Pull-out or suture anchor	End-to-end suture
Zone IIb	Camper's chiasm injury	Rupture	End-to-end suture or no repair	End-to-end suture
Zone IIc	Rupture	Rupture	End-to-end suture or no repair	End-to-end suture

The sample size was not determined based on statistical power since this study is exploratory in nature. We aim to represent all injury types (a total of five classifications) based on the revised classification system. Eight fingers will be sutured for each classification. Assuming successful surgery in seven out of eight fingers (87.5% success rate), the confidence interval was estimated to be 0.646-1.104. The repair success rate was confirmed to fall within a realistic accuracy range.

The final determination of the sample size accounts for the feasibility and technical applicability of the proposed endoscopic flexor tendon repair technique, in addition to the feasibility of conducting research using cadavers and medical resource constraints. Therefore, the sample size was deemed reasonable.

Furthermore, the traditional zone classification [15] for flexor tendon injuries was modified in this study, and a new classification system was introduced. When devising the endoscopic flexor tendon repair technique, we found that the traditional classification system did not adequately reflect the selection of treatment methods based on injury type and damage site. Particularly, anatomical structures and surgical field limitations required consideration, leading to the development of a new classification. The revised classification system accounts for differences in the surgical field during endoscopic procedures, the type of tendon to be repaired, and the appropriate repair methods for each injury type.

Outcomes

In this study, the soft tissues and tendon sheath will be dissected after endoscopic tendon repair to expose the suture site. We will then evaluate the primary outcome and secondary outcomes (2) and (3).

Primary Outcome

The primary outcome is the success rate of endoscopic tendon repair. The success rate is an important indicator of the surgical method’s appropriateness and feasibility of the technique. A high success rate suggests the reliability and safety of the procedure, indicating its potential for future clinical applications. In contrast, a low success rate highlights the need for improvements in the technique and aids in identifying areas requiring further refinement. The criteria for failure are suture failure or excessive gap formation between the tendon ends.

Secondary Outcome

Secondary outcomes include (1) suturing time, (2) gap distance between tendon ends, (3) occurrence of complications from the suture, (4) a new classification of tendon injuries that considers endoscopic tendon suturing (Figures 6, 7, Table 1), and (5) surgical technique video.

Regarding the occurrence of complications from suturing (3), we will monitor for nerve injury, arterial injury, and incomplete tendon end suturing. Moreover, any unexpected complications that arise will be documented.

The surgical technique video (5) will document the steps of the endoscopic tendon repair procedure, aiming to provide a clear and educational resource for other surgeons. The video will demonstrate the developed technique, including key steps and any specific considerations required when performing the procedure. It will be designed as an instructional tool to aid surgeons in effectively replicating the method in their practice.

Statistical analysis

Regarding the primary outcome, the success rate of repairs will be reported as the percentage of successful repairs.

Regarding secondary outcomes, the mean suturing time and the gap between tendon ends will be reported with 95% confidence intervals. The complication rate will be presented as a percentage of the total number of repairs.

SPSS software (version 28.0.0.0; IBM Corp., Armonk, NY) will be used for all statistical calculations. Statistical significance will be set at p-values <0.05.

## Discussion

This IDEAL-D Stage 0 (Preclinical) study [10] advances endoscopic tendon repair for Zone 1 flexor tendon injuries, addressing the soft tissue damage and restricted joint ROM caused by open repair [5]. Combining a case report and a protocol, it establishes the feasibility of a novel MIS technique.

The case report confirmed successful one-loop suturing in one cadaver, validated by a 5 N tensile test with no neurovascular damage (Figure 4, Video 1). Prolonged suturing time, however, highlighted the need for specialized instruments. The protocol builds on this, evaluating the technique across five cadavers (40 fingers) to quantify suturing time, assess complications, and validate a new classification system (Figure 5, Table 1). This system modifies the traditional zone classification [15] by incorporating endoscopic-specific criteria, such as tendon stump accessibility, with feedback from five hand surgeons collected via structured interviews to enhance clinical applicability.

Endoscopic repair minimizes incision size and tissue damage, potentially improving ROM compared to open surgery [7-9]. While prior studies localized tendons endoscopically [7], complete suturing, as achieved here, is novel. However, endoscopy requires specialized skills, and inadequate visualization may compromise success. The protocol quantifies these challenges, informing refinements like adopting a triple-loop suture in Stage 1 (Idea) for greater tensile strength, as the case report’s one-loop suture showed limited stability under prolonged manipulation [17,18].

Cadaveric studies provide technical consistency but cannot assess healing, limiting clinical outcome predictions. The protocol’s suturing time data may not fully reflect clinical performance. The sample size of five cadavers ensures coverage of the new classification system, aligning with Stage 0’s exploratory goals. Future Stage 1 studies will test the technique in a small patient cohort, assessing safety and initial efficacy, followed by Stage 2a (Development) for refinements and Stage 2b (Exploration) for multicenter validation.

This Stage 0 study, with its case report and protocol, demonstrates endoscopic repair’s potential, paving the way for minimally invasive hand surgery under the IDEAL-D framework.

## Conclusions

This study introduces an innovative approach to flexor tendon repair that minimizes tissue invasion through the use of endoscopy, which may represent a significant advancement in the field of tendon surgery. However, several challenges remain, owing to the nature of research conducted on cadavers. Moving forward, we will conduct research focused on clinical applications, in accordance with the IDEAL-D framework.
